# Effects of androgens and rutting season on drug metabolizing enzymes in dromedary camels

**DOI:** 10.1590/1984-3143-AR2019-0119

**Published:** 2020-05-20

**Authors:** Aziza Thamer El-Khaldi, Abdelgadir Musa Homeida

**Affiliations:** 1 Department of Biology, College of Science and Basic and Applied Scientific Research Center, Imam Abdulrahman Bin Faisal University, Dammam, Saudi Arabia

**Keywords:** drug metabolizing enzymes, androgens, poll gland, rutting camel

## Abstract

A series of experiments were conducted to investigate the effect of rutting season on metabolism of testosterone (T) and its effect on drug metabolizing enzymes in dromedary camels. Serum and tissue samples were collected from liver, testes and poll glands of rutting and non- rutting camels treated with T at a dose of 0.5 mg/kg or 5α-dihydrotestosterone (DHT) at a dose of 0.2 mg/kg, given intramuscularly for 7 days. Liver samples were also used to monitor drug metabolizing enzymes. Testosterone and DHT concentrations were significantly (*P*<0.05) increased in testicular tissue and peripheral circulation of rutting camels compared to non-rutting camels and in non-rutting camels treated with T or DHT. Drug metabolizing enzymes of phase-1 reaction were significantly (*P*<0.05) inhibited in livers of rutting camels and in non-rutting camels treated with T and DHT. It is suggested that co-administration of drugs metabolized by oxidation with androgens should be avoided. Such drugs may cause adverse drug reaction in rutting camels.

## Introduction

Dromedary camel is a multipurpose domestic animal that remains central to the subsistence and socioeconomic livelihoods of pastoralists in desert, semi desert and tropical regions. Drugs are extensively used in camels to save the life of animal, to restore the normal function of body organs and to improve production and quality of cameline products. Drug manufactures give no specific recommendations for the camel. Therefore, the doses used clinically in this species are in general extrapolated from doses recommended for other large domestic animals. This is not without danger because toxic effects sometimes occur in camels which are given certain drugs at doses apparently harmless to other species (Al-Dughaym et al., 1998; Ali and Hassan, 1986; Homeida et al., 1981).The increased susceptibility of camels to certain drugs may at least be partly explained by the comparatively low drug metabolizing enzyme activities in this species (El Sheikh et al., 1986, 1988). Hepatic phase-1 drug metabolizing enzymes are responsible for metabolism of numerous xenobiotics and endogenous substances. The metabolite, when compared to parent compound, is less active, less lipid soluble and highly ionizable polar compound which is readily excreted (Brander et al., 1982). The effect of sex hormones on phase-1 oxidative metabolism has been documented in humans (Bruchovsky and Wilson, 1968), goats (Witkamp et al., 1990) and cattle (Janus and Antoszek, 1999).

Seasonal breeders like camels (Ali et al., 2018) have dormant phases of reproductive cycle, which could suggest potential difference in drug pharmacokinetic response due to different levels of sex hormones. The androgens concentrations in blood of camels were increased during rutting season far higher than in bulls and were correlated with radical changes in behavior of the animal (Yagil and Etzion, 1979). These androgenic effect in camels were associated with hyperactivity of occipital glands (poll glands) during rutting season (Yagil and Etzion, 1980). This study was conducted to investigate the effect of dromedary rutting season on activity of drug metabolizing enzymes.

## Materials and methods

### Animals and treatments

Twenty-four mature healthy male camels (*Camelus dromedarius*) aged 7-8 years and weighing 500-600 kg were used. Animals were kept under nomadic conditions in an open yard during the breeding season (Nov-Feb) or non-breeding season. They were fed daily with 2 kg of mixture of barley and wheat bran, and hay and water were provided *adlibitum*. All the experimental procedures described below were approved by the University Ethics Monitoring Committee and carried out in accordance with Guidelines of Animal Welfare committee. Camels were allocated to 1 of 4 experiments, each containing 6 animals as follows:

**Experiment 1.** Rutting camels (*n*=6) were slaughtered at mid breeding season to obtain tissues from testes, liver and occipital (tubulo- alveolar) glands (Poll glands) situated at the occipital region between the two ears. The glands were active during this time secreting a dark brown material. Tissues were placed in liquid nitrogen. Jugular blood from all animals in different experiments was obtained by venipuncture, centrifuged at 1500g, and serum was stored at -30 °C until analysis;
**Experiment 2.** Non-rutting camels (*n*=6) during non-breeding season were slaughtered to obtain samples from testes, liver and poll glands;
**Experiment 3.** Non-rutting camels (*n*=6) were used to study the effect of testosterone on activity of drug metabolizing enzymes. Testosterone (Organon, Oss, The Netherlands) in sesame oil was administered intramuscularly (i.m.) at a dose of 0.5 mg/kg/day for 7 days. Animals were then slaughtered to obtain testes, liver and poll glands;
**Experiment 4.** Non-rutting camels (*n*=6) were used to study the effect of 5α –dihydrotestosterone (DHT) on activity of drug metabolizing enzymes. DHT (Sigma, England) in sesame oil was administrated i.m. at a dose of 0.2 mg/kg/day for 7 days. Animals were slaughtered to obtain tissues.

### Drug metabolizing enzyme activity

All operations were carried out at 4 °C. Pieces of liver were homogenized in ice cold isotonic KCl solution (0.15M, pH 7,4) by 6-8 stroked in a motor-driven Teflon homogenizer for one minute to give 28% W/V liver homogenates. The crude homogenates were then centrifuged at 4 °C for 10 minutes at 10000 x g. The microsome rich fractions were decanted and used for measuring the activities of the enzymes. A microsomal fraction was prepared for the estimation of protein concentrations as described elsewhere (El Sheikh et al., 1988; Mazel, 1971). Enzyme activities were estimated by methods previously described and validated (El Sheikh et al., 1986; Homeida et al., 1993), briefly. aminopyrine N-demethylase was estimated by measurement of formaldehyde following the procedure of Nash (1953) as described by Mazel (1971). Aniline 4-hydroxylase was determined by measuring the quantity of p-aminophenol produced as described by Mazel (1971). UDP-glucanosyltransferase activity was estimated as described by Dutton and Storey (1962), using O-aminophenol as substrate. Cytochrome *P*-450 concentrations was estimated by spectrophotometer according to Omura and Sato (1964) as described by Mazel (1971). The method depends on the combination of the reduced form of cytochrome *P*-450 with carbon monoxide to form a complex with an absorption maximum at 450nm and a minimum at 405nm. All enzymatic reactions were conducted at 37 °C under air at conditions of initial velocity with appropriate blanks. The incubation time was 30 min for aminopyrine N-demethylase and cytochrome P-450 and 20 min for aniline 4-hydroxylase and UDP-glucuronyl-transferase.

### Hormonal measurements

Samples from male subjects were analyzed using a testosterone ELISA assay kit (Assay Designs, Enzo Life Sciences, U.K.). Samples were diluted (1:10) and run in duplicate. This kit has a lower limit of detection of 5.67 pg/mL, an inter-assay coefficient of variation (CV) of 11.3% and an intra-assay CV of 10.0% The cross reaction was testosterone 100%, 19-hydroxytestosterone 14.6%, androstenedione 7.20%, dehydroepiandrosterone 0.72%, estradiol 0.40% and <0.001% for dihydrotestosterone, estriol, aldosterone, corticosterone, cortisol, cortisone, estrone, progesterone. Sample 5α-dihydrotestosterone was determined by ELIZA kit (Novus Biologicals Europe, Abingdon, UK). The detection limit was 6 pg/mL, inter-assay CV was 8.5%, and intra assay CV was 6.95%. The cross reaction was dihydrotestosterone 100%, testosterone 8.7%, 5β-dihydrotestosterone 2.0%, androstenedione 0.2%, and < 0.01% for progesterone, esterone, estradiol and cortisone.

### Data analysis

Effects of season or treatment on enzyme activity, and hormonal concentrations were analyzed by analysis of variance (ANOVA). Where effects of treatment were significant, mean differences were examined with Duncan's multiple range test (Duncan, 1955). Data are presented as means ±SD and differences were considered significant at *P*< 0.05.

## Results

Liver protein concentration were similar in rutting and non-rutting camels. No statistically significant changes were observed in liver protein of camels treated with testosterone or DHT. The activity of drug metabolizing enzymes is shown in Table 1.

**Table 1:** Mean (±SD) protein concentration and activity of drug metabolizing enzymes in livers of rutting and non-rutting camels treated intramuscularly with testosterone (0.5 mg/kg) or dihydrotestosterone (0.2 mg/kg).

**Variable**	**Rutting camels (*n*=6)**	**Non-rutting camels**		
		**Untreated controls (*n*=6)**	**Treated with testosterone (*n*=6)**	**Treated with DHT (*n*=6)**
Protein (mg/g): whole homogenate	151.86±2.12a	154±3.11a	156.3±2.15a	153.6±2.41a
Cytosolic fraction	90.6±1.96a	93.3±2.12a	92.4±1.85a	91.7±1.91a
Microsomal fraction	22.09±0.361a	24.1±0.251a	23.2±0.41a	24.4±0.26a
Enzyme activity: Cytochrome P-450 (n mole reduced cytochrome-Co/mg/mg microsomal protein/min)	0.111±0.003a	0.223±0.016b	0.123±0.002a	0.116±0.002a
Amino pyrine-N-demethylase (n moles of formaldehyde/mg microsomal protein/min)	2.3±0.061a	6.00±0.53b	4.1±0.75c	2.2±0.06a
Aniline-4- hydroxylase (n moles of p-aminophen ol/mg microsomal protein/min)	0.211±0.011a	0.416±0.042b	0.36±0.041c	0.231±0.011a
UDP-glucuronyl transferase (n moles of O-aminophenol/mg microsomal protein/min)	0.890±0.023a	0.940±0.025a	0.905±0.061a	0.896±0.031a

Means in each row, values with different letter are significantly different *P*<0.05.

Activity of cytochrome P-450, aminopyrine-N-demethylase and aniline-4-hydroxylase in rutting camels were significantly (*P*<0.05) lower than in non-rutting camels. Activity was significantly (*P*<0.05) inhibited by testosterone and DHT treatment. DHT treatment was more potent (*P*<0.001) than testosterone in inhibiting the oxidative enzymes. UDP- glucuronyl transferase activity was not affected in rutting or following treatment with androgenic hormones. Tissue and peripheral serum concentration of testosterone and DHT (Table 2) was significantly (*P*<0.05) higher in rutting than in non-rutting camels.

**Table 2:** Mean (±SD) serum and tissue concentrations of testosterone and 5α - DHT in serum and tissue of liver, testes and poll gland of camels.

**Variable**	**Rutting camels (*n*=6)**	**Untreated control (*n*=6)**	**Non-rutting camels**	**Treated with DHT (*n*=6)**
			**Treated with testosterone (*n*=6)**	
Testosterone: Testes (ng/g)	320.6±9.6a	1.96±6.6b	220.1±7.2c	160±5.6b
Liver (ng/g)	0.36±0.04a	0.41±0.031a	1.5±0.31b	0.46±0.03a1b
Poll Gland (ng/g)	120.6±4.5a	0.66±0.021b	0.71±0.03b	4.6±0.20c
Serum (ng/mL)	2.86±0.081a	1.2±0.20b	2.5±0.31a	1.1±0.12b
DHT: Testes (ng/g)	150.2±4.7a	62.3±2.1b	101±3.1c	72.1±2.3b
Liver (ng/g)	7.7±0.51a	0.53±0.01b	2.2±0.20c	3.1±0.4c
Poll gland (n/g)	90.5±1.45a	0.41±0.32b	6.2±0.51c	18.4±1.2b
Serum (ng/mL)	1.41±0.33a	0.31±0.03b	2.3±0.41a	4.6±0.80c

Means in each row, values with different letter are significantly different, *P*<0.05.

Administration of testosterone and DHT to non-rutting camels caused increased concentration of the hormones of tissues and serum.

## Discussion

In the present study, and during the breeding season, the rutting camel was characterized by having a higher level of testicular and peripheral serum testosterone and DHT concentration compared to non-rutting camels in line with previous findings, that the concentration of testosterone in testicular tissue (Berndtson et al., 1983; Johnson and Thompson, 1987) and in plasma (Ali et al., 2018; El-Harairy and Attia, 2010; Nasr and El-Azab, 1990; Yagil and Etzion, 1980) was increased during breeding season than in non-breeding season. However, these authors didn't measure DHT in their camels.

The increased secretion of these androgens may be as a result of increase in Leydig cell number per testes (Johnson and Thompson, 1987) or increase in volume of interstitial tissue (El-Harairy and Attia, 2010) during the breeding season or both.

The results also show that the metabolism of androgen was increased during breeding season. Testosterone is rapidly metabolized to estradiol (aromatizing pathway) or to 5α -DHT (5α -reductase pathway) (Bruchovsky and Wilson, 1968; Celotti et al., 1979). It is likely that higher activity of 5α - reductase was expressed in testes and poll glands and consequently, higher concentration of DHT was produced in rutting camels. The fact that 5α -DHT is more potent than testosterone (Godfrey et al., 2004) in biological test and in eliciting behavioral response suggests that testosterone metabolism could be a necessary step in expression of hormonal effects (Homeida and Cooke, 1984; Massa and Martini, 1974) . Histological examination of the poll gland during the rutting season showed that the gland may be endocrine in nature (Tingari et al., 1984). During this time the gland actively displays strong α-smooth muscle actin, S-100 protein and cytokeratin immunoreactivity (Ebada et al., 2012), suggesting a contractile and secretory activity (Donato et al., 2013; Hinz et al., 2001). However, from the present data, it seems likely that the glands accumulate and extensively metabolize testosterone to DHT. Further research is needed to confirm the endocrine nature of the glands. One effect of this high production of androgens was inhibition of oxidative drug metabolizing enzymes in rutting camels. Indeed, administration of testosterone and DHT to non-rutting camels have inhibited the activity of oxidative drug metabolizing enzymes. Likewise, administration of testosterone to goats have produced an inhibition of mixed function oxidase (Nouws et al., 1988).

Cows proved to be more active metabolizers of phase-1 metabolism than bulls (Janus and Antoszek, 1999). However, in rats, androgenic hormones were inducers of cytochrome P-450 (Gustafsson et al., 1982), suggesting that the extent and direction of sex dependence drug metabolism varies from one species to another (Witkamp et al., 1990).

The activity of phase-2 hepatic enzyme UDP-glucuonyl transferase remained unchanged in rutting and in non-rutting treated with testosterone or DHT, suggesting that androgen effects on drug metabolism is limited to phase-1 oxidation.

## Conclusion

In conclusion, results obtained indicate that breeding season lead to activation of poll gland with increased α -reductase activity and excessive 5α -DHT production. The androgens have produced inhibition of phase-1 oxidative enzyme and may impair the elimination of drugs that undergo metabolism by these enzymes, that could eventually lead to adverse drug reaction in the rutting camel.
